# Design process of cementless femoral stem using a nonlinear three dimensional finite element analysis

**DOI:** 10.1186/1471-2474-15-30

**Published:** 2014-02-03

**Authors:** Mohd Yusof Baharuddin, Sh-Hussain Salleh, Ahmad Hafiz Zulkifly, Muhammad Hisyam Lee, Alias Mohd Noor, Arief Ruhullah A Harris, Norazman Abdul Majid, Ab Saman Abd Kader

**Affiliations:** 1 Department of Biomedical Engineering, Faculty of Engineering, University of Malaya, Kuala Lumpur, Malaysia; 2 Centre for Biomedical Engineering Transportation Research Alliance, Universiti Teknologi Malaysia, Skudai, Johor, Malaysia; 3 Department of Orthopaedic, Traumatology & Rehabilitation, Kuliyyah of Medicine, International Islamic University Malaysia, Kuantan, Pahang, Malaysia; 4 Department of Mathematical Sciences, Faculty of Science, Universiti Teknologi Malaysia, Skudai, Johor, Malaysia; 5 Transportation Research Alliance, Universiti Teknologi Malaysia, Skudai, Johor, Malaysia

**Keywords:** Morphology, Femur, Hip replacement, Cementless hip, Finite element analysis

## Abstract

**Background:**

Minimal available information concerning hip morphology is the motivation for several researchers to study the difference between Asian and Western populations. Current use of a universal hip stem of variable size is not the best option for all femur types. This present study proposed a new design process of the cementless femoral stem using a three dimensional model which provided more information and accurate analysis compared to conventional methods.

**Methods:**

This complete design cycle began with morphological analysis, followed by femoral stem design, fit and fill analysis, and nonlinear finite element analysis (FEA). Various femur parameters for periosteal and endosteal canal diameters are measured from the osteotomy level to 150 mm below to determine the isthmus position.

**Results:**

The results showed better total fit (53.7%) and fill (76.7%) canal, with more load distributed proximally to prevent stress shielding at calcar region. The stem demonstrated lower displacement and micromotion (less than 40 μm) promoting osseointegration between the stem–bone and providing primary fixation stability.

**Conclusion:**

This new design process could be used as a preclinical assessment tool and will shorten the design cycle by identifying the major steps which must be taken while designing the femoral stem.

## Background

Total hip arthroplasty (THA) is recognized as the most successful orthopaedic surgery in the 20^th^ century. This fact was confirmed by the United Nation (UN) and World Health Organization (WHO) which declared the years 2000 – 2010 as the “Bone and Joint Decade” due to the rise of musculoskeletal diseases aligned with the aging population [1]. In the United States alone, the demand for THA is estimated to rise by 174% by 2030 to 572 000 procedures [2]. The high prevalence of hip arthroplasty has encouraged implant manufacturers to produce better designs with optimized fixation as prescribed by the orthopaedic surgeon. However, there is no universal design for hip implants which could fit and fill all femur types [3-5]. Noble et al. [3] classified the endosteal canal into three different shapes based on the canal flare index (CFI): stovepipe shape (CFI < 3.0), normal shape (3.0 < CFI < 4.7) and champagne flute shape (CFI > 4.7). In our previous study [6-9], we found that Asian medullary canals are categorized under normal and champagne flute shape. This peculiar proximal hip morphology of Asian’s requires the development of an appropriate design which could prevent complications due to the implant’s geometric mismatch such as stress shielding, micromotion and loosening [8]. However, most commercial stems are designed and manufactured in Europe and North America which are tailored to their anatomical structure [6-8]. This development led to the design of a femoral stem which suits local population morphology and lessens bone loss during surgery. This newly designed stem distributes loads optimally and attains better primary fixation. Furthermore, the rapid growth of in silico methods aids in shortening the design process for implant manufacturers and researchers prior to clinical trial.

Several studies have been performed regarding the cementless femoral stem using in silico and experimental methods [10-12]. Dopico – Gonzalez et al. [10] presented a robust tool for probabilistic finite element analysis of the cementless hip focusing on femur characteristics and implant design geometry between Proxima short stem and IPS stem showing good agreement with the in-vitro study. In addition, Pettersen et al. [11,12] supported the excellent correlation between an actual human cadaver and finite element study while investigating the feasibility of subjects specific to stress shielding and micromotion using cementless Summit stem. Ando et al. [13] also performed a finite element analysis to compare their stems for Japanese dysplastic hip (FMS and FMS-anatomic) with other commercial stems such as Omnifit, Omniflex and IDS focusing on contact stress, relative motion and load transfer prior clinical use. The results showed the load was transferred mostly in proximal region with low micromotion value, which supported the excellent success rate of this implant [14,15]. Furthermore, Rawal et al. [16] manufactured the Indian femoral stem using 3 axis CNC machine after finding that the equivalent von Misses stress result from finite element analysis was below 160 MPa to prevent the endosteal fracture. In this study, we apply a similar method using nonlinear three dimensional finite element analysis in the design process of a cementless stem for Malays. Finite element analysis became a useful tool for researchers to predict early and medium term results [17]. Furthermore, identifying the problem and rectifying it using finite element analysis helped to significantly improve the implant during the design process before actual clinical trials. Promising results from the analysis point towards less dependency on in vivo experiments. We believe that finite element analysis using the actual femora might become a useful tool for pre-clinical testing of newly designed implants. The finite element could become a “safety measure” for new stems before the clinical trial. If the stem fails at this stage, the stem would most probably fail clinically. This study aims to propose a design process of the cementless femoral stem which began by reverse engineering the three dimensional morphology analysis, design of optimal fit and fill femoral stem, and analysis using finite element to rectify its disadvantages before fabrication.

## Methods

**Figure 1:**
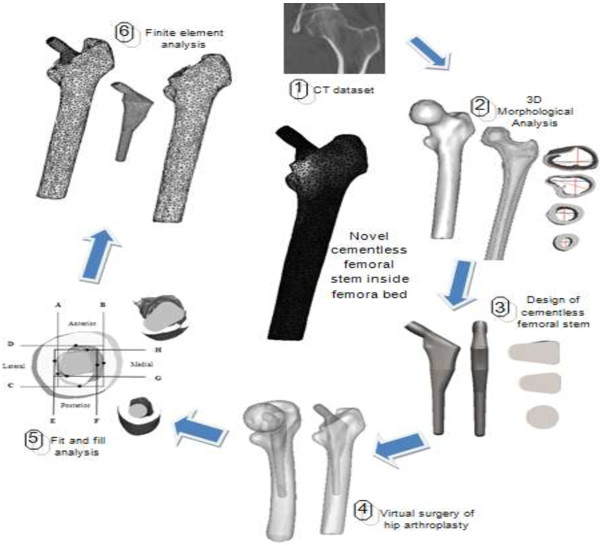
Summarize steps of designing the cementless hip arthroplasty.

**Figure 2:**
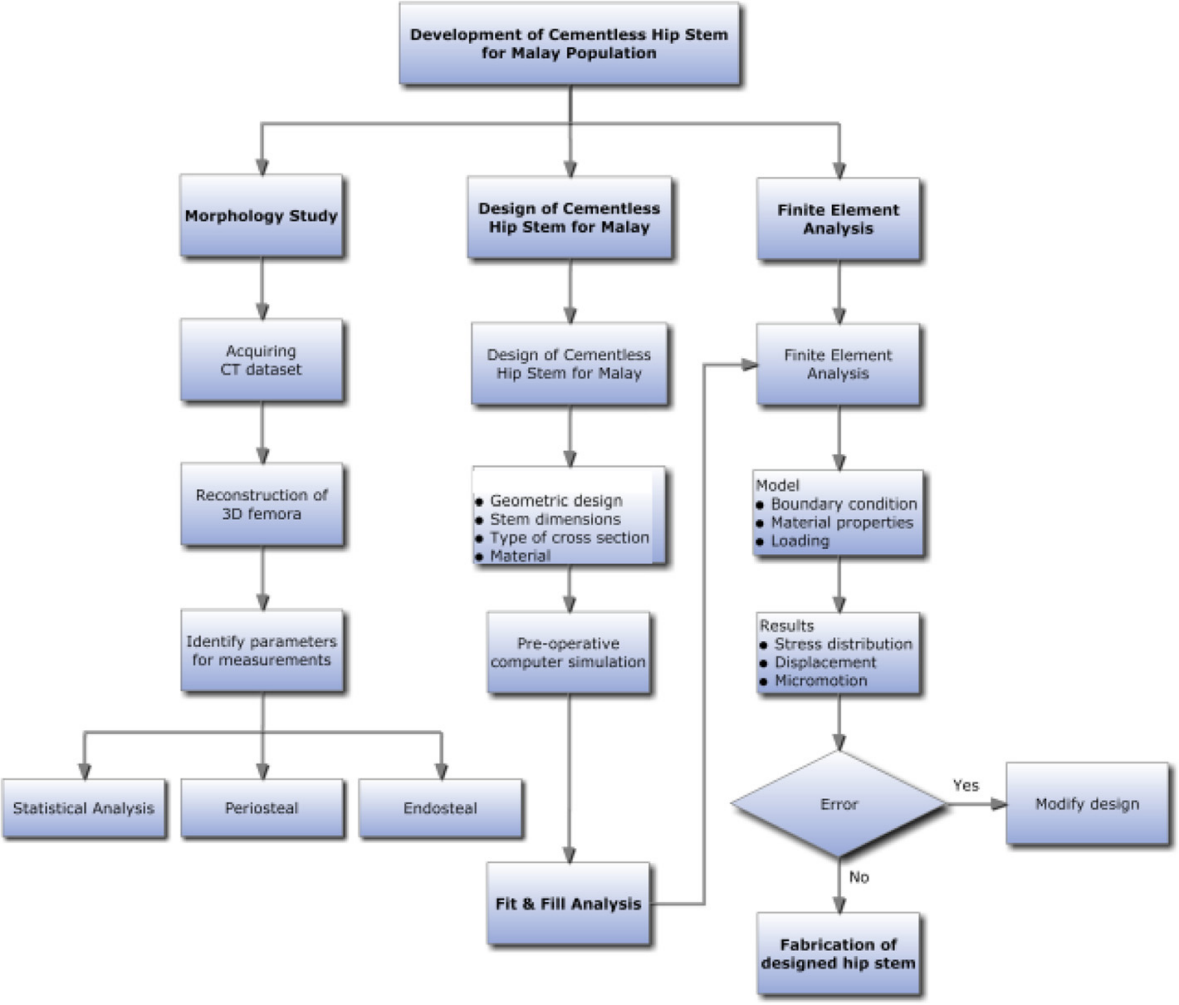
Proposed new design process framework of the cementless femoral stem.

The steps and framework of a cementless femoral stem design are summarized in Figures 1 and 2. Generally, the femur image of the patient is taken by standard radiograph and the template given by the implant’s manufacturer was used to determine the implant’s size. However, we proposed another method which was more efficient than the conventional methods. The first step was to acquire the computed tomography (CT) dataset of the femur, followed by the reconstruction of three dimensional (3D) morphological analysis. The 3D anthropometric dataset of 60 healthy subjects were then used to design a cementless femoral stem before performing a ‘virtual surgery of hip arthroplasty’ using the “averaged” femora based on our previous studies [6-9]. The canal fit and fill were analyzed for the optimal implant, and finally the finite element model was analyzed to examine stress distribution, displacement and micromotion.

### Three dimensional (3D) morphological analysis

A cross sectional study was carried out from January 2009 to December 2009 following approval from the National Medical Research Register (NMRR) and the local hospital ethics committee. The procedure was approved by Universiti Teknologi Malaysia Human Ethics Research Committee, and the written informed consent was filled by participant prior study. We measured the femora periosteal and endosteal canal diameters of 60 healthy femora (30 male, 30 female). The average age for all subjects was 25.01 ±5.18 years. The average weight was 70.76 ± 14.38 kg for male and 53.31 ± 13.11 kg for female. The average height was 170.96 ± 6.37 cm for male and 156.02 ± 6.17 cm for female. Subjects were excluded from this study if they were pregnant, had experienced prior femur injury or bone disease (osteoporosis, osteoarthritis, and rheumatoid arthritis), had abnormal body mass index (BMI), wore implants or underwent a computed tomography scan less than 6 months from the date of consent filling. This was verified by clinical examination and computed tomography (CT) images. The femora image was acquired using four row multi slices CT scanner (Somatom, Volume Zoom, Siemens) operating at 120 kV and 90 mAs. Other scanning parameters were set: 1.25 mm collimation, 3.0 mm thickness, 1.5 mm recon increment, 12.0 mm table feed per rotation and 512 x 512 pixel resolution. Subjects were asked to lay down in a supine position with their feet stabilized using the specially designed wooden jig to standardize foot position during image acquisition. Gonad shields were used and no contrast media was administered.

**Figure 3:**
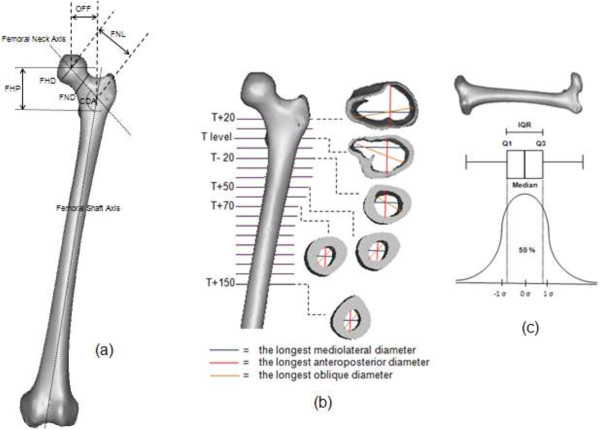
Three-dimensional (3D) morphological analyses of the femur (a) periosteal (b) endosteal (c) interquartile range (IQR) descriptive analysis.

The three dimensional (3D) femora was reconstructed by importing the CT images into Mimics 12.1 software (Materialize, Leuvan, Belgium) as shown in Figure 3. CT image thresholds were classified to distinguish compact bone and spongial bone after checking the profile line through the cross section CT gray slice. Threshold profile was set to 662-1988 HU for compact bone and 148-661 HU for spongial bone [18]. Femora mask was computed into a 3D model and orthogonally cut into a few sections after measuring 10 mm intervals from the osteotomy level to 150 mm below lesser trochanter, T. The 3D sliced femora were converted into stereo lithography model for accurate measurement using commercial CAD software (SolidWorks 2009 SP2.1, Dassault System, Massachusetts, USA). The longest mediolateral, anteroposterior and oblique medullary canal diameters were measured for each slice. The smallest endosteal canals in mediolateral directions were computed with consideration of the isthmus level. In addition, the medial and lateral cortex radius, and medial and lateral tapered angles were also measured from mediolateral view. Other definitions used in this study are shown below:

a) Collo diaphyseal angle (CDA) – angle between femur neck axis and femur shaft axis.

b) Femoral neck length (FNL) – distance between femur head center and intersection point of femur shaft axis and femur neck axis.

c) Femoral head offset (OFF) – horizontal distance of femur neck length.

d) Femoral head diameter (FHD) – maximum diameter of the femur head.

e) Femoral neck diameter (FND) – minimum diameter of the femur neck.

f) Femoral head position (FHP) – vertical distance between femur head center and center of lesser trochanter.

g) Anteversion – angle between femur neck axis and line connecting two posterior condyles in transverse view.

h) Canal flare index (CFI) – ratio of the endosteal canal diameter at osteotomy level slice (20 mm above lesser trochanter) and isthmus level. CFI classified femora into 3 shapes: stovepipe (< 3.0), normal (between 3.0 and 4.7) and champagne-flute (> 4.7) [3].

The datasets were statistically analyzed with SAS 4.3 software (SAS Institute Inc., Cary, NC, USA). The comparison of the morphological analysis between our study and other populations [3,16,19-22] are shown in Table 1. The value α ≤ 0.05 was set to determine whether the data were statistically significant [6-9]. Normality assumption for each group of data was verified using Kolmogorov – Smirnov method [6-9]. Folded F method was used to examine the equality of data variances if the data was normally distributed. The probability was then checked using *t*-test either by Pooled method or Satterthwaite method, according to the equality of the variance. If the data was not normally distributed, nonparametric one-way ANOVA was adopted using Wilcoxon scores. The probability value was then determined by Chi-Square values through Kruskal-Wallis test. In addition, descriptive statistics and inter quartile range (IQR) were used to design the stem profile [6-9].

**Table 1:** Comparison of the femur morphology with other populations

**Parameters**	**Our Study (Malay)**	**Mahaisavariya et al. (Thai)**[19]	**Rawal et al. (Indian)**[16]	**Bo et al. (Japan)**[20]	**Noble et al. (Caucasian)**[3]	**Massin et al. (France)**[21]	**Rubin et al. (Swiss)**[22]
	**(n = 60)**	**(n = 108)**	**(n = 98)**	**(n = 100)**	**(n = 80)**	**(n = 200)**	**(n = 32)**
Collo diaphyseal angle (^o^)	130.46 ± 4.02	128.04 ± 6.14	124.42 ± 5.49	137.40 ± 4.80	125.40	123.10 ± 8.2	122.90 ± 5.76
Femoral head offset (mm)	30.35 ± 4.26	-	40.23 ± 4.85	31.50 ± 5.00	-	41.00 ± 6.20	47.00 ± 7.20
Femoral neck length (mm)	45.30 ± 4.74	46.22 ± 5.14	48.40 ± 5.66	-	-	49.40 ± 6.80	-
Femoral head diameter (mm)	40.81 ± 3.43	43.98 ± 3.47	45.41 ± 3.66	-	45.90	45.60 ± 4.20	43.40 ± 2.26
Femoral neck diameter (mm)	28.95 ± 3.37	-	-	-	-	-	-
Femoral head position (mm)	53.14 ± 4.87	48.94 ± 4.95	52.33 ± 3.19	-	-	58.70 ± 7.20	56.10 ± 8.20
Anteversion (º)	19.10 ± 8.67	11.37 ± 7.65	10.90 ± 4.22	27.00 ± 14.10	10.00	-	-
Medial cortex radius (mm)	71.77 ± 30.59	-	100.00	96.70 ± 26.80	-	-	-
Lateral cortex radius (mm)	82.90 ± 40.81	-	80.00	96.10 ± 23.50	-	-	-
Medial tapered angle (º)	3.18 ± 2.53	-	4.00	3.80 ± 1.70	-	-	-
Lateral tapered angle (º)	16.45 ± 3.52	-	4.00	3.70 ± 1.60	-	-	-
AP T + 20 width (mm)	31.12 ± 3.70	-	26.26 ± 3.70	-	-	-	-
ML T + 20 width (mm)	44.05 ± 4.59	-	36.78 ± 5.32	-	51.50	44.10 ± 6.00	43.10 ± 5.20
AP T-20 width (mm)	17.54 ± 2.93	-	15.36 ± 2.37	15.00 ± 2.30	-	-	-
ML T-20 width (mm)	18.45 ± 2.93	-	16.20 ± 2.71	18.00 ± 3.00	-	19.60 ± 2.90	21.00 ± 2.70
Isthmus position (mm)	112.83 ± 11.80	112.93 ± 17.96	107.80 ± 9.73	73.00 ± 18.90	116.40	-	105.70 ± 17.90
AP Isthmus width (mm)	13.12 ± 2.46	-	11.47 ± 2.11	10.40 ± 2.60	-	-	-
ML Isthmus width (mm)	9.73 ± 1.80	10.50 ± 1.81	9.02 ± 1.92	11.90 ± 2.60	12.00	12.40 ± 2.30	13.10 ± 2.10
Canal flare index	4.65 ± 0.83	-	4.23 ± 2.97	-	-	3.60 ± 0.80	3.36 ± 0.75

**Notes:** Data are presented as mean ± SD.

*Abbreviations: AP* anteroposterior, *ML* mediolateral, *T* center of lesser trochanter, ° degree.

### Philosophy behind stem design

**Figure 4:**
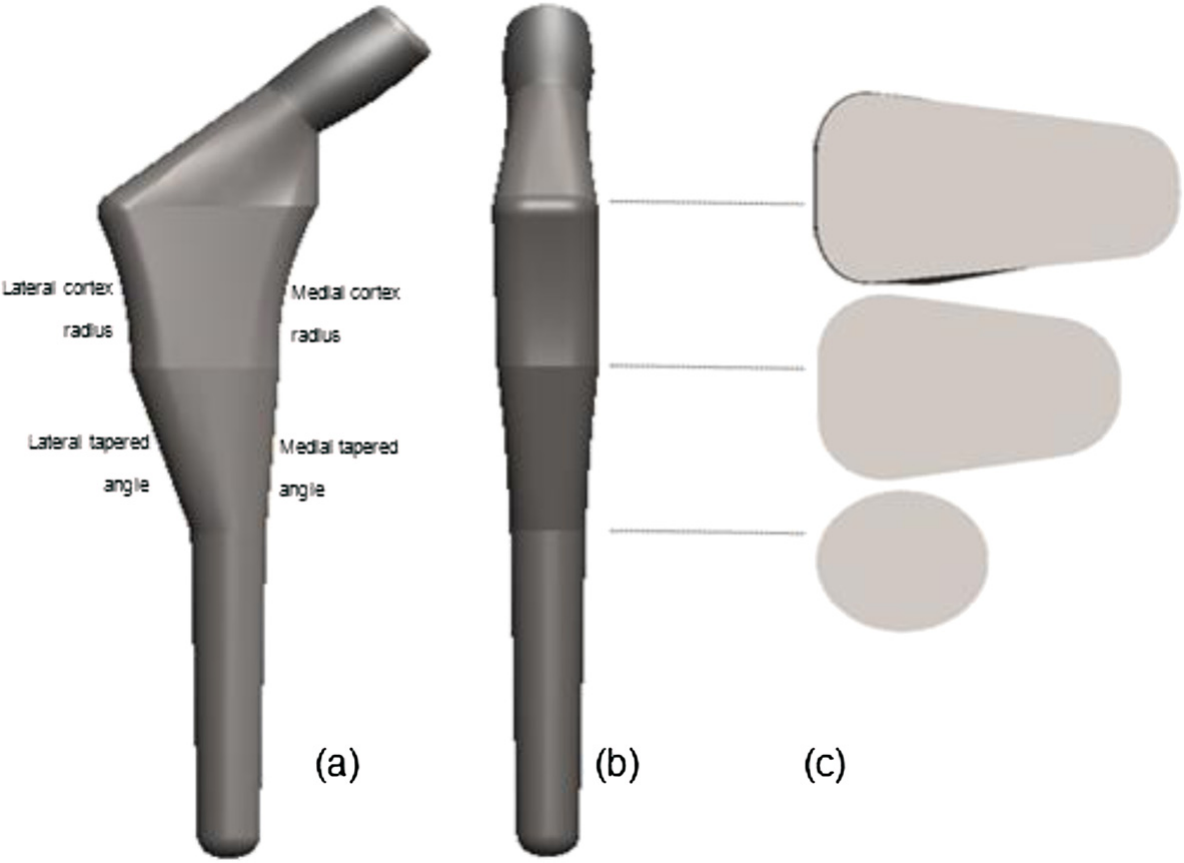
Cementless femoral stem design according to the femur morphology (a) mediolateral view (b) anteroposterior view (c) cross section view.

In general, custom made stems are used for their shape which is designed in line with the patient’s hip morphology [23]. However, manufacturing costs and time consumed are major topics of discussion within the orthopedic community. As standard, off the shelve implants do not cater to all types of femora, a few modifications from the implant’s manufacturer have been done, especially on size and the metaphyseal region. Kaya et al. [24] reported the modification of Anatomic Medullary Locking (AML) stem (Depuy, Warsaw, IN, USA) especially at the metaphyseal, called medial modified aspects (MMA) due to narrower and shorter hips of Japanese. The AML-A was made from cobalt chromium with porous coating circumferentially at proximal region. Another question was whether the smaller size implant solved the problem or was the profile used as a guideline not similar to the Asian femora. We have computed the descriptive statistics for each parameter used in the stem design profile from the dataset as shown in Table 2. In addition to mean, minimum, maximum, and range values, we also reported inter quartile range (IQR) values as a resistance statistic toward outliers compared to the range and standard deviation. The “average” morphology could be used as a guideline for the implant’s design, which better addresses the population diversity. The assumptions made regarding size selection and implant design were based on this “average” femur provides the actual figure of the bone itself. The best fit and fill were considered to contribute to the fixation stability of the implant. Combining the parameters acquired from periosteal and endosteal canal, the stem design was done carefully as shown in Figure 4. The basic principles were used in the design are as follows:

a) The implant width is in accordance with the femora endosteal canal diameter to achieve optimal fit and fill with the bone and promoting osseointegration between implant – bone.

b) The implant length and distal size did not exceed the position of the isthmus level or the isthmus canal diameter.

c) The stem neck followed standard 12/14 taper.

d) The optimal stem cross section geometry is according to the endosteal canal shape; wedge shape at the metaphyseal region, tapered at middle region, and cylindrical at distal.

e) The medial and lateral curvature followed the actual femora proximal radius; lateral flares provide the “rest fit” for the implant, better physiological load and prevent subsidence distribution.

f) The proximal region provides three contact points between the implant and bone for better primary fixation stability.

g) The distal stem was designed straight with 1° taper to reduce strain distally.

h) The safety factor was greater than 1.0. If the safety factor is less than 1.0, the material has yielded and the design is not safe.

i) The computer simulations displayed no stress shielding or larger von Mises stress than intact femora [16], along with acceptable displacement and micromotion (< 40 μm).

**Table 2:** Descriptive statistics using interquartile range (IQR) analysis for stem design profile

**Parameters**	**Lower Quartile**	**Upper Quartile**	**IQR**	**Mean**	**Minimum**	**Maximum**	**Range**
Collo diaphyseal angle (^o^)	126.67	132.78	6.11	130.68 ± 1.68	126.68	132.78	6.10
Femoral head offset (mm)	27.29	33.09	5.80	30.55 ± 1.53	27.30	33.06	5.76
Femoral head position (mm)	48.56	57.15	8.59	52.72 ± 2.45	48.76	57.12	8.36
Medial cortex radius (mm)	50.19	95.35	45.16	65.84 ± 13.61	50.50	95.20	44.70
Lateral cortex radius (mm)	52.56	95.95	43.39	74.07 ± 10.17	52.95	95.47	42.52
Medial tapered angle (º)	1.14	4.36	3.22	3.74 ± 3.40	0.47	12.80	12.33
Lateral tapered angle (º)	14.70	19.00	4.30	16.78 ± 1.24	14.76	18.84	4.08
AP T + 20 width (mm)	28.66	32.95	4.29	31.12 ± 3.70	26.05	43.90	17.85
ML T + 20 width (mm)	40.92	47.41	6.49	44.05 ± 4.59	33.83	53.47	19.64
AP T-10 width (mm)	18.44	22.33	3.89	19.99 ± 0.98	18.53	22.20	3.67
ML T-10 width (mm)	20.13	24.67	4.54	22.35 ± 2.97	15.77	29.43	13.66
AP T-40 width (mm)	11.71	15.55	3.84	13.41 ± 1.02	11.80	15.54	3.74
ML T-40 width (mm)	12.13	14.84	2.71	13.73 ± 3.11	9.19	19.29	10.10
Isthmus position (mm)	100.00	120.00	20.00	112.86 ± 8.42	100.00	120.00	20.00
AP Isthmus width (mm)	10.95	14.51	3.56	13.23 ± 3.36	9.01	21.10	12.10
ML Isthmus width (mm)	8.29	10.89	2.60	9.58 ± 0.82	8.32	10.87	2.55

*Abbreviations: AP* anteroposterior, *ML* mediolateral, *T* center of lesser trochanter, ° degree.

### Fit and fill analysis through virtual hip arthroplasty surgery

**Figure 5:**
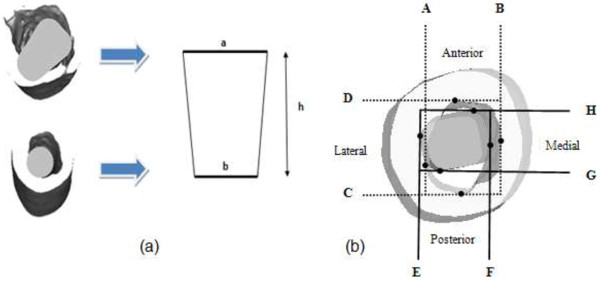
Fit and fill analysis (a) contact area (fit) substitute as trapezoid’s base, and h, slice height (b) canal fill as the ratio between implant (EF x GH) and endosteal canal (AB x CD).

Virtual implantation was simulated in Mimics 12.1 software where the newly designed cementless femoral stem was aligned inside the “average” femur canal which was developed from the previous morphological study as shown in Figure 5. The femur neck was resected at the osteotomy level (20 mm above the center of lesser trochanter). The fit and fill were essential to the femoral fixation stability and determined the success of the implant. Fit was defined as 1 mm or less perpendicular distance between the implant and endosteal [20,25]. The fit was determined by translating the contact between the implant and the endosteal canal into a straight line [20]. The contact area was computed by trapezoid area, with fit acting as the base of the trapezoid, and the height from the cross section as shown in Figure 5(a). In addition, fill was defined as the percentage of the implant area within the femur intra medullary [5,20] from the anteroposterior view and mediolateral view for each cross section as illustrated in Figure 5(b). The cross section was divided into three levels; proximal, middle and distal. The proximal represented the metaphyseal region, medial (the upper end of isthmus) and distal (10 mm above the stem’s tip). The goal was to acquire the optimal fit and fill and as such, the stem should be within reasonable size, making it easier for the orthopedic surgeons without breaking the femur. The newly designed stem was then compared with other cementless stems [20]. The Fukui Medical School (FMS) stem was straight, coated at one third proximal, had proximal lateral flare with different medial radius, and was designed specifically for Japanese dysplastic hips. Later, they designed FMS-A which followed the anatomic femora and introduced the anterior flare. On the other hand, the Omniflex and Omniflex-J was a straight cylindrical stem tapered at one third distal. The AML and Harris-Galante stem was straight stem, coated with a porous surface and sintered titanium fiber composite pad, respectively. In addition, the IDS stem was purposely designed for optimal fit and fill in endosteal canal.

### Nonlinear 3D finite element analysis

The implant model was designed using computer aided design (CAD) software (SolidWorks 2009 SP2.1, Dassault System, Massachusetts, USA). As the ultimate goal was to achieve optimal fit and fill, the stem was designed according to the anthropometric study done prior [6-8]. The geometric was carefully chosen based on the cross section of the femora which provides optimal contact area between the stem and endosteal canal to promote osseointegration. This feature was simply the decipherable criterion of the stem design. The stem was then imported to finite element software (Abaqus 6.8, Dassault System, Massachusetts, USA) in geometric data file format (.igs) to convert the triangle node mesh into the tetrahedral node mesh, and to produce better mesh by repairing the edges. The model input file (.inp) was then converted into stereo lithographic (.stl) format using finite element software (Marc.Mentat, MSC Software, Santa Ana, CA). Although there were many ways to convert three dimensional stem models into surface meshing, the author found out that this procedure produces better meshing for this study. The mesh created directly through the automatic meshing routine from Marc.Mentat software, for example, created irregular mesh size and density even though a uniform mesh could be produced using this software by treating the model based on the surface plane, and repairing the elements manually by MAGICS (embedded in Mimics software).

The cementless femoral stem was next aligned within the femora canal to simulate hip arthroplasty, and the stem neck was positioned at an anteversion angle in relation to normal femora. The osteotomy level was set to 20 mm above the center of the lesser trochanter. These two models were in surface mesh to form the stem and endosteal canal contact. A perfect contact fit between stem and endosteal was assumed by creating the ‘virtual surgery femora’ from the surface mesh of the cementless stems’ correct position during the surgery through similar to Boolean operation in MAGICS. The redundant elements resulting from this operation were manually removed and repaired especially at the boundary region between the endosteal and implant. The femora surface mesh on the top covering the lateral shoulder implant was also removed mimicking the reaming procedure. This ‘virtual surgery femora’ and stem were subsequently converted into solid linear first order tetrahedral elements in Marc.Mentat software. These models were constructed into a refined mesh, and mesh convergence study was performed on the femoral stem to determine the optimum number of meshes. An average of 13 200 elements with 4200 nodes was found to be optimal for the cementless femoral stem. The size of the element mesh for femur and implant was set to 0.4 mm. The convergence testing was performed using the auto switch relative for residual forces or displacements with 0.1 tolerances. The tetrahedral mesh generated on Marc.Mentat software took approximately 30 minutes and the analysis took a further hour to complete. We used a Lenovo workstation with Intel® Pentium® microprocessor and 4 GB of RAM for all modeling and analysis in this study. Convergence study is essential to ensure that the result is independent of the mesh density and not underestimated. The intact femora consisted of 5000 nodes and 34 000 elements, and the ‘virtual surgery femora’ consisted of 7900 nodes and 41 900 elements. The material properties of the cementless femoral stem were assigned as titanium alloy (Ti_6_Al_4_V) with Young’s Modulus of 110 GPa and a Poisson’s ratio of 0.3 [13]. In addition, the femora were assumed as isotropic and linear elastic, with bone properties determined according to the CT datasets grey level values. The correlation proposed by Carter and Hayes between the modulus of elasticity of the bone, E and its apparent density was used in this study as shown in equation 1 [26]. The cancellous and cortical bones are assumed merely at different ends of a continuum spectrum.

(1) $$E=3790\;\rho^{3}$$

**Figure 6:**
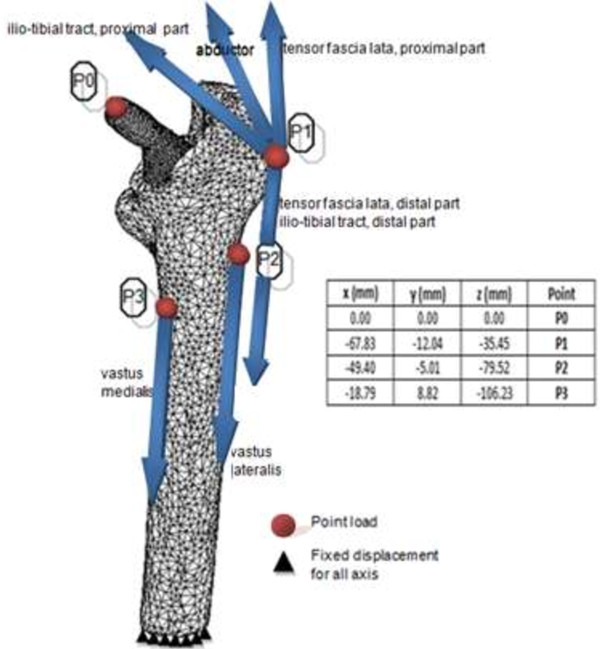
Muscles point load configuration in physiological loading.

The finite element model was completely restrained distally. Two static physiological loadings were simulated; normal walking and stair climbing. Kassi et al. [27] pointed out that the stair climbing load is more detrimental than single stance; however, Petterson et al. [11,12] demonstrated that in general both physiological loadings showed similar stress shielding patterns when finite element data was compared with the experimental data. In this study, femora configuration and muscle attachment with loading directions were based on the extensive studies of Bergmann and Heller et al. [28,29] as illustrated in Table 3 and Figure 6 based on a body weight of 700 N. A deformable to deformable contact was created between stem and femora. Viceconti et al. [30] indicated that the best friction coefficient correlation between the experimental and simulation study was between 0.2 – 0.5. This was supported by Rancourt et al. [31] who determined the friction coefficient experimentally as 0.4. In addition, Abdul-Kadir et al. [32] used 0.4 to validate the finite element with experimental data while studying the effect of interference fit for primary hip stability. Based on these previous studies, a friction coefficient for both models was set at 0.4 in this study. Another parameter involved was the smoothing of one step function in Coulomb friction model known as *SL* in Marc.Mentat software which significantly influenced the micromotion [32]. Shirazi-Adl et al. [33] pointed that the non-linearity between medullary canals – stem was high attributing to the micromotion threshold (150 mm) prior to slip load which anticipated by Coulomb model. Based on this Coulomb model [32,33], the *SL* was set to 0.1.

**Table 3:** Physiological loading condition for normal walking (top) and stair climbing (bottom)

**Normal Walking**				
**Muscles point load (N)**	**x**	**y**	**z**	**Point load**
Hip contact	-378	-229.6	-1604.4	P0
Abductor	406	30.1	605.5	P1
Tensor fascia lata, proximal part	50.4	81.2	92.4	P1
Tensor fascia lata, distal part	-3.5	-4.9	-133	P1
Vastus lateralis	-6.3	129.5	-650.3	P2
**Stairs Climbing**				
**Muscles point load (N)**	**x**	**y**	**z**	**Point load**
Hip contact	-415.1	-424.2	-1654.1	P0
Abductor	490.7	201.6	594.3	P1
Ilio-tibial tract, proximal part	73.5	-21	89.6	P1
Ilio-tibial tract, distal part	-3.5	-5.6	-117.6	P1
Tensor fascia lata, proximal part	21.7	34.3	20.3	P1
Tensor fascia lata, distal part	-1.4	-2.1	-45.5	P1
Vastus lateralis	-15.4	156.8	-945.7	P2
Vastus medialis	-61.6	277.2	-1869.7	P3

The micromotion algorithm used in this study had been validated experimentally in house [32]. This algorithm was written in Compaq Visual Fortran software (Compaq Computer Corporation) as the subroutine to compute and demonstrate micromotion in finite element software (Marc.Mentat). The first subroutine computed the nodal displacement between the interface of the cementless femoral stem and endosteal canal, while the second subroutine automatically stored the interfacial nodes differences. As both stem-femora shared the similar nodes prior, the differences due to micromotion were shown as the contour plot in the post-processing result. The result focused on three parameters; equivalent von Mises stress, micromotion and displacement. Two other parameters were observed in this study; total strain energy density and contact normal stress.

## Results

### Three dimensional (3D) morphological analysis

The comparison between the parameters in different populations was depicted in Table 1. We compared our data with Thai, Indian, Japan, Caucasian, French and Swiss populations. The collo diaphyseal angle for Malays was higher (130.46º) compared to other populations except Japanese. However, due to the small physique of the Malay population, smaller sizes had been anticipated in several parameters such as femoral head offset, femoral neck length and femoral head diameter. There is a 16.65 mm difference between Malay and Swiss populations in terms of femoral head offset which was a crucial parameter in determining the size of the hip stem during pre-operative planning. The canal flare index (CFI) for our study was classified as normal shape which is within the range of 3.0 - 4.7 [3]. Our study showed that 52% were classified as normal shape femora, and 48% champagne-flute shape femora. No stovepipe shape femora was found in our study but was observed in other populations. The mean and standard deviations of endosteal canal diameters in mediolateral (ML) and anteroposterior (AP) directions at different levels were tabulated in Table 1. The western population has larger endosteal diameter values compared with our study except at the metaphyseal region which demonstrated small differences. The endosteal enlargement rate (interval 10 mm for each slices) showed the highest value at the metaphyseal section which was up to 20 mm above zero level, T. The femora cavity enlargement gradually decreased at the diaphyseal region. We found statistical significance (*p* < 0.05) between genders for isthmus position and 81.67% femora had the isthmus position within 100 to 120 mm from T.

### Novel cementless femoral stem design

The new stem was designed according to the morphological analysis as shown in Figure 4. However, we found that using the average value exclusively did not represent the whole population. Descriptive statistics by inter quartile range (IQR) method is tabulated in Table 2. The average medial cortex radius was set to 65.84 mm which is suitable for femora within a range of 50.19 – 95.35 mm. We have also introduced the lateral flare which shows a 74.07 mm radius that correlates with the “rest fit” and load distribution to the stem. The wedge shape was chosen as the best cross section at the metaphyseal level. This shape mimics the actual endosteal canal, and provides optimal fit and fill. On the other hand, the middle region of the femora endosteal demonstrated a tapered figure in mediolateral view. The medial taper was determined at 3.74°, and lateral taper at 16.78°. In addition, the distal parts were assumed cylindrical straight. The femoral head offset was set to 30.55 mm horizontally, and 52.72 mm vertically. Our study showed smaller offset compared with western populations which generally used standard off-shelf stem sizing of 35 – 37.5 mm, with collo diaphyseal angle of 135°. Finally, the femoral stem was also shorter as the stem’s length is associated with the isthmus position.

### Three dimensional (3D) fit and fill analysis

**Figure 7:**
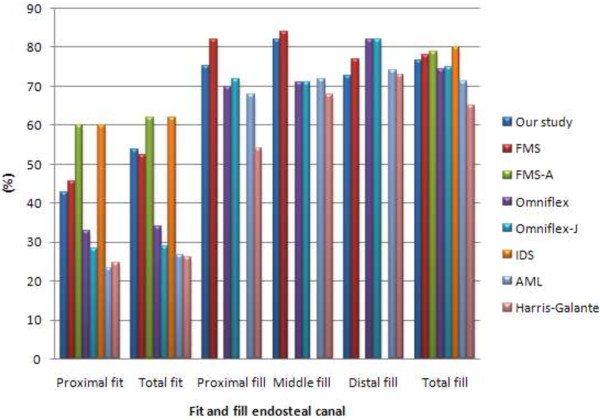
Comparison of fit and fill analysis between different cementless stem.

The analysis for fit and fill as shown in Table 4 divides the femora into three regions; proximal, middle and distal. Total fit for this newly designed stem was 53.70%, with a 42.90% proximal part contact area between stem – endosteal. The implant fills the endosteal canal to a high percentage; 83.10% at anteroposterior view and 71.70% at mediolateral view. The comparison between different cementless stems [13,20] is illustrated in Figure 7. This newly designed stem was among the highest percentage of fit and fill compared to others except the Fukui Medical School (FMS and FMS-A), and IDS stem. These three stems also applied the same principle which is to obtain optimal fit and fill. However, the Omniflex and Omniflex-J showed the highest canal fill at distal with 82%.

**Table 4:** Fit and fill analysis between stem and endosteal canal

**Level**	**Fit (%)**	**Anteroposterior view**			**Mediolateral view**		
		**Femur (mm**^**2**^**)**	**Implant (mm**^**2**^**)**	**Fill (%)**	**Femur (mm**^**2**^**)**	**Implant (mm**^**2**^**)**	**Fill (%)**
Proximal	42.89	873.42	843.29	96.55	1108.56	600.00	54.12
Middle	58.18	586.19	505.35	86.21	604.49	470.10	77.77
Distal	62.65	934.52	621.90	62.19	747.46	621.90	83.20
Total	53.68	-	-	83.10	-	-	71.70

### Nonlinear three dimensional (3D) finite element analysis

**Figure 8:**
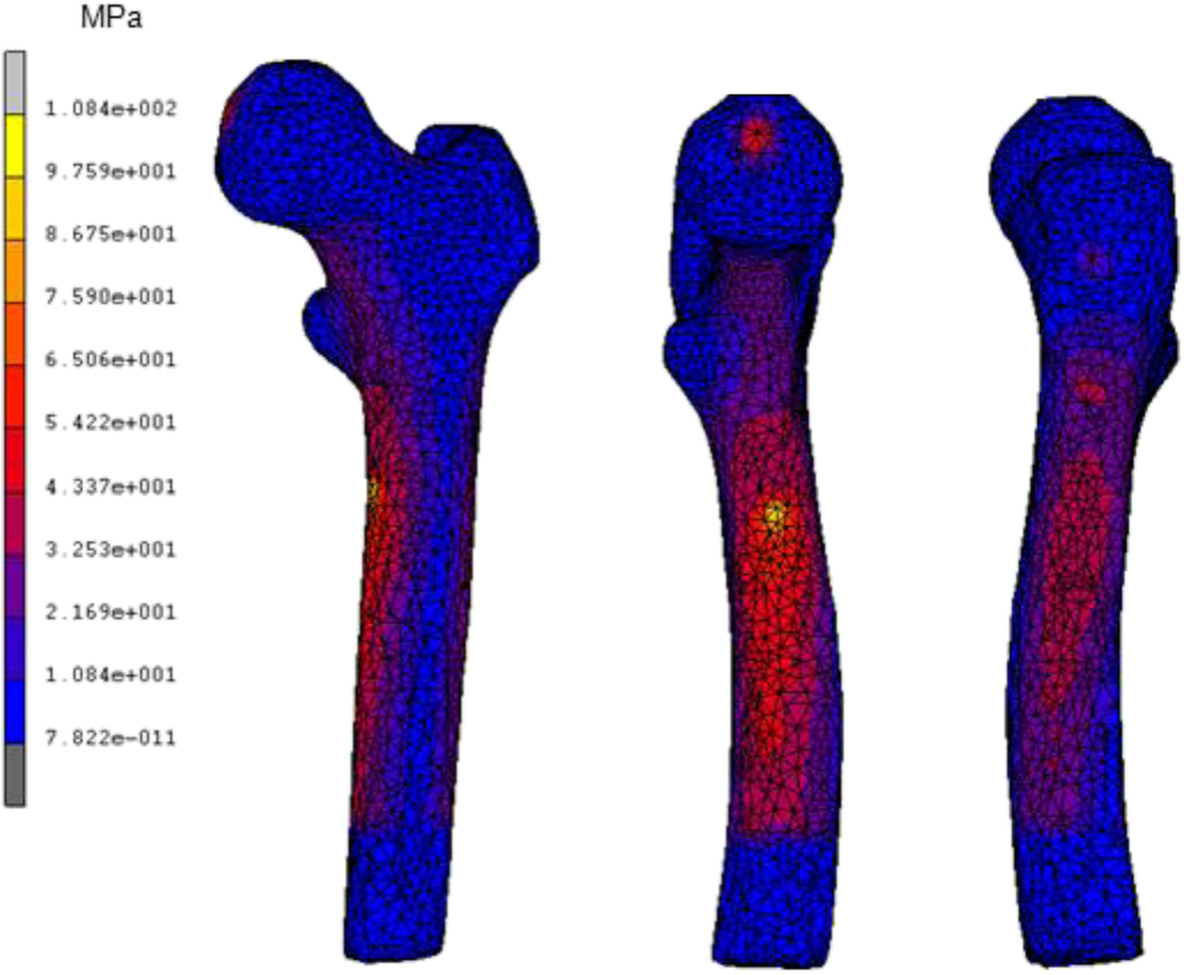
Contour plots of equivalent von Mises stress using stair climbing loading from (a) frontal view, (b) medial view and (c) lateral view.

**Figure 9:**
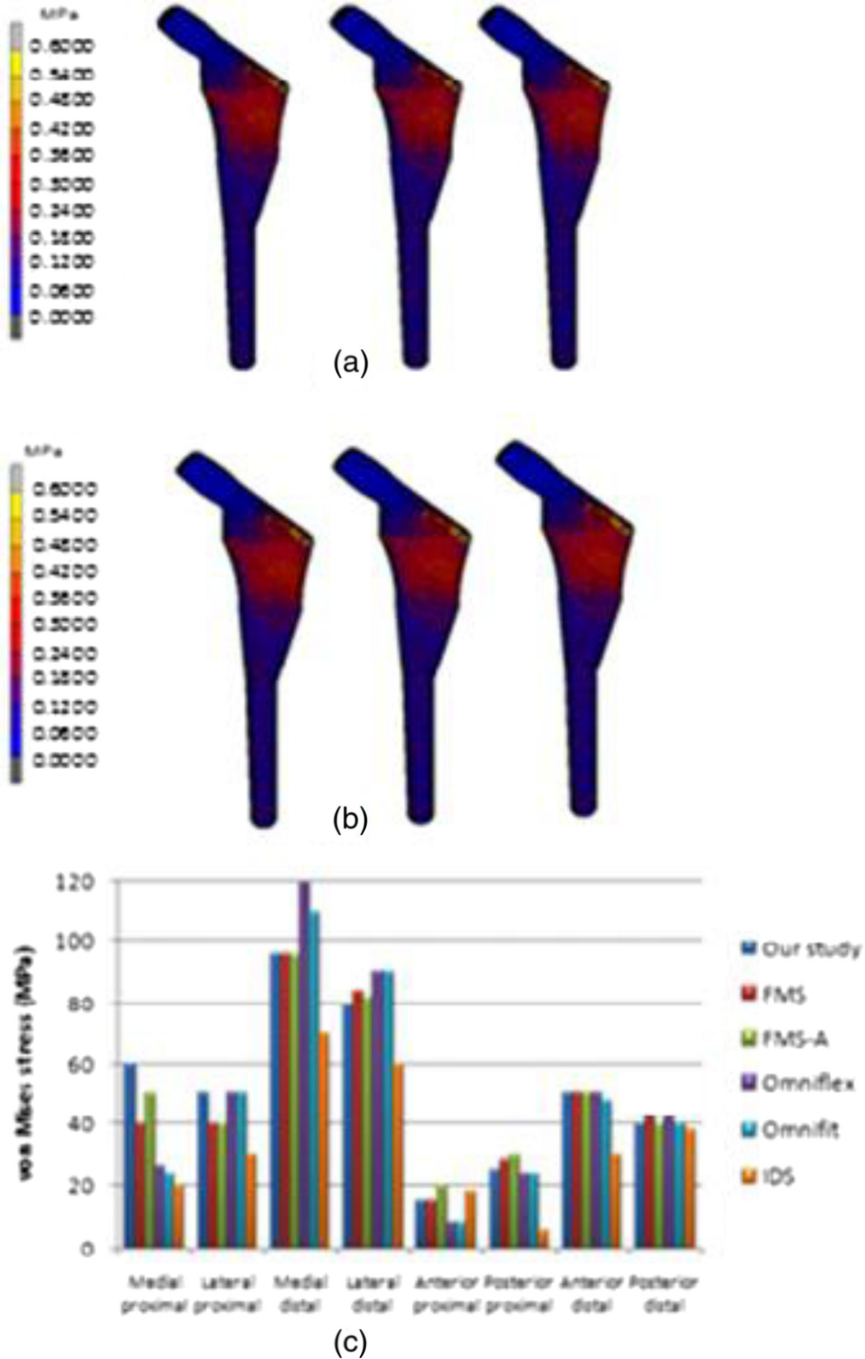
Contour plots of equivalent von Mises stress using (a) normal walking loading and (b) stair climbing loading, (c) comparison with other implants in normal walking loading at different cortical positions.

**Figure 10:**
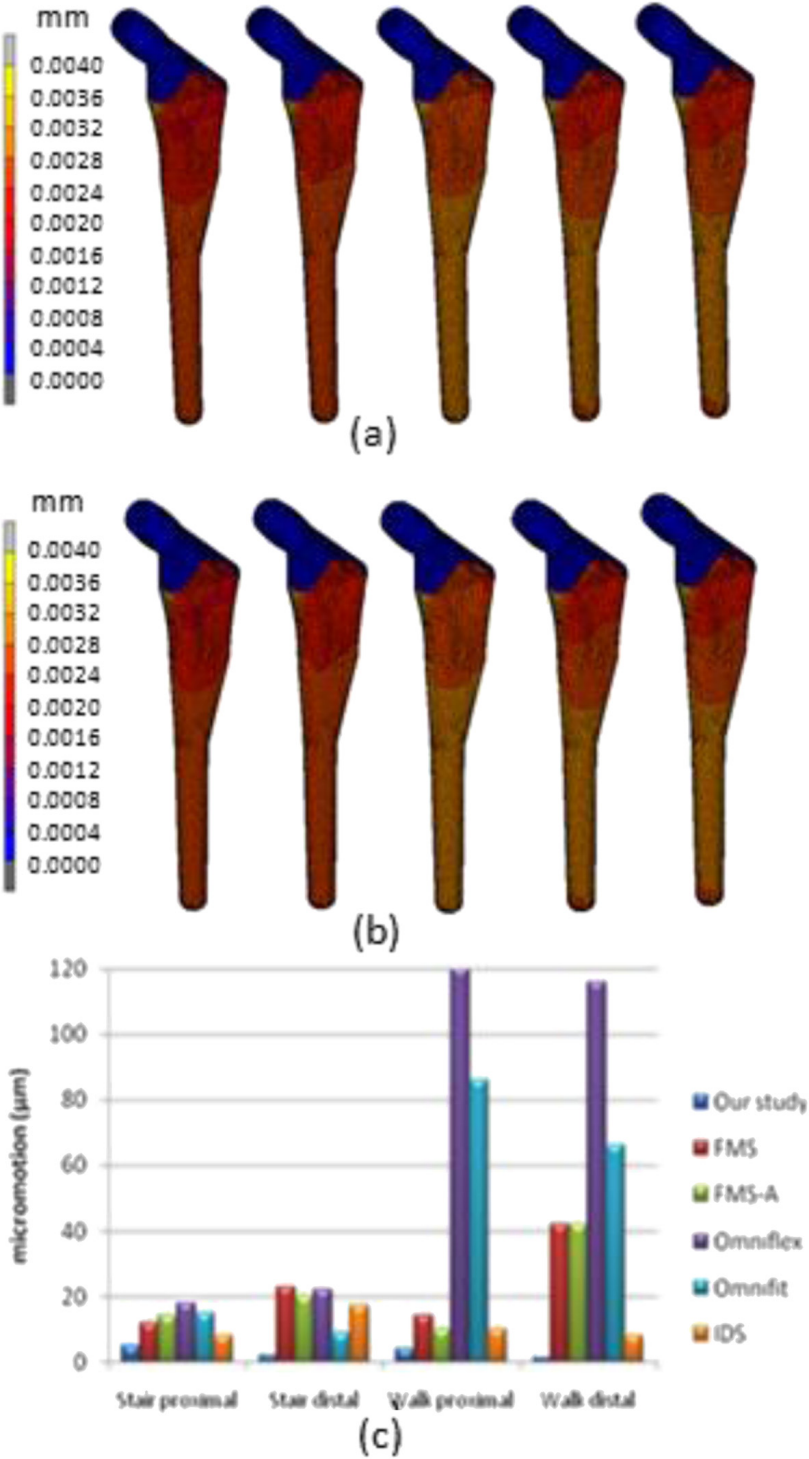
Contour plots of micromotion using (a) normal walking loading and (b) stair climbing loading, (c) comparison with other implants in both physiological loadings.

**Figure 11:**
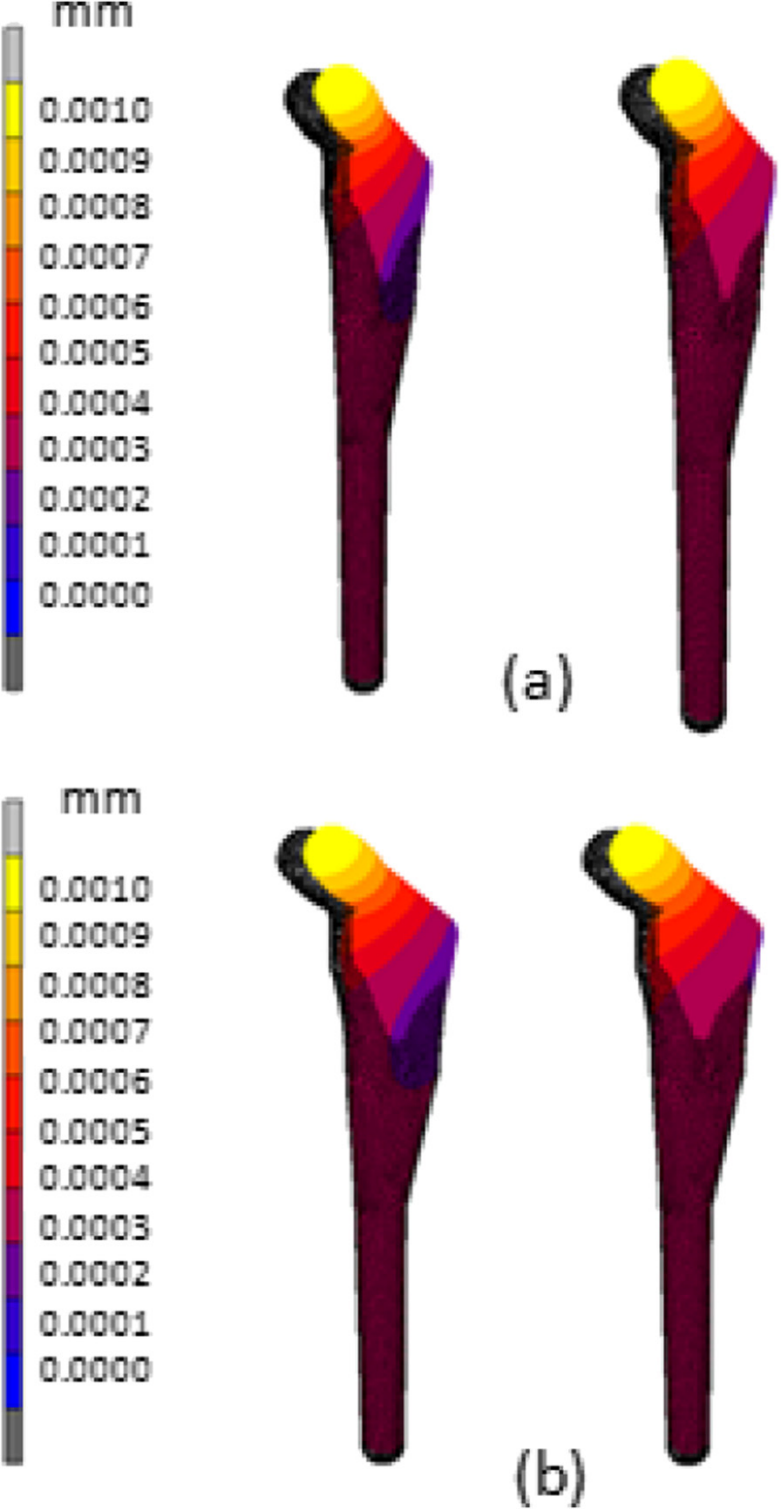
**Contour plots of displacement using (a) normal walking loading and (b) stair climbing loading.** Color contour represented the deformed shape after physiological loading and pink mesh represented the original location.

The von Mises stress distribution for intact femur using the stair climbing load demonstrated in Figure 8 showed the maximum stress of 108.4 MPa at medial calcar. This study used both types of physiological loading; normal walking and stair climbing. However, the result did not illustrate significant differences. The maximum stress observed was 65.38 MPa at the proximal region and minimum stress was 1.28 x 10^-12^ MPa at distal region as shown in Figure 9. When the limit was scaled to 600 000 Pa, we found that the stress was normally distributed at metaphyseal region which is essential for stability fixation, and to prevent stress shielding at the proximal level. The safety factor for this newly design stem was computed as 2.45. The comparison between other cementless stems at different cortical bone positions [13,20] showed that our newly designed stem was not inferior to other femoral stems. Our study showed highest stress at medial calcar with 60 MPa which indicated stress shielding did not occur at this region as shown in Figure 9 (c). The micromotion and displacement contour plots were shown in Figures 10 and 11. As these parameters are closely related to the promotion of bone osseointegration, we found that the maximum value for micromotion was 4.76 μm, and a displacement of 1.34 μm. Our study demonstrated the lowest micromotion compared to other femoral stems in physiological loading ranging from 1.5 – 5.0 μm as shown in Figure 10(c). This ensured that osseointegration occurred between the bone – stem interface, and fibrous tissue formation was prevented which reflected the implant’s fixation stability. We also noted the maximum total strain energy density of 1.31 kJ/m^3^, and contact normal stress of 28.95 MPa.

## Discussion

### Three dimensional (3D) morphological analysis

Morphological analysis was the first stage prior to designing the cementless femoral stem. We reconstructed the 3D femur model from the CT datasets. Several studies affirmed the accuracy of this method compared to other medical imaging modalities [4,16,34]. The 3D model provided more information on the morphology, which reflected correct stem sizing and bone condition during the pre-operative planning [34]. On the other hand, standard x-ray images were reported as inaccurate compared to CT images due to distortion and measurement errors using 2D view [22,25,34]. Still, CT images showed several limitations such as the slice thickness of 2-5 mm and slice spacing of 10 mm, measurement error due to partial volume effect, beam hardening, and patient movement [22]. Thus, 3D model was the best choice in designing the implant which mimics the actual femora; periosteal and endosteal canal. The threshold was determined based on the grey slice images through the profile line to distinguish the transition zone between cortical bone and cancellous bone [5,18].

To develop a cementless stem, accurate measurement was essential as the stem’s design required optimal fitting and filling within the femur canal. In addition, understanding the morphology enhanced primary stem fixation stability especially at the metaphyseal region, and supported maximum physiological loading to the femora [14,20]. Walker et al. [35] pointed to the importance of proximally fitting, and regarded the femoral stem below lesser trochanter as not crucial with the lateral flare cementless anatomical stem. Furthermore, it would generate compressive force between femur head and greater trochanter [36]. Several studies have introduced indices that represent the endosteal canal, especially the metaphyseal region [3-5,21]. Noble et al. [3] classified femur intramedullary shape with the canal flare index (CFI); ratio of endosteal canal width at osteotomy level and isthmus level. On the other hand, Husmann et al. [4] suggested several flare indices as a new anatomic category with detailed characteristics of the zone capital for uncemented implants. Laine et al. [5] illustrated another index at the metaphyseal region to distinguished the variety of proximal intramedullary shapes. Furthermore, Massin et al. [21] highlighted the necessity of this region and produced a series of the monoblock stems based on the sufficient filling in of the frontal plane.

We have compared the femora morphology between our study and other populations as tabulated in Table 1. The femoral head offset between our study and western populations differed by 10 – 17 mm. In general, the implant was designed utilizing western anthropometric database as the gold standard which varied in linear and angular specifications [8,37]. The implants produced were bigger in size, risking more bone stock in endosteal through surgery [8,37,38]. This phenomenon has led to the tendency on the part of global implant manufacturers’ to produce smaller implants with other modifications. Conversely, the declination of collo diaphyseal angle (CDA) with 1° causes the acetabular cup to incline by 0.45° and to antevert by 2° [39]. The commercial stem utilized 135° as the universal design, and this resulted in a shorter femoral head offset that required restoration with trochanteric osteotomy [21]. The offset was crucial to ameliorate hip stability, providing an enhanced range of motion (ROM) of abduction and improving abductor strength [21,40,41]. Elhadi et al. [42] pointed out a variance of 3.5 mm lower in offset due to anteversion while using 2D and 3D measurement methods. The anteversion could vary by 22° - 50° causing distortion by standard x-ray [4,42], and 60% of cases reported that the offset was not restored using the universal stem design while 31% used the 131° design [43]. Still, Elhadi et al. [42] found no correlation between these two parameters as the periosteal feature was independent compared to endosteal.

### Three dimensional (3D) fit and fill analysis and femoral stem design

Several studies showed that fit and fill play an important role in implant fixation stability [13,20,44]. However, the optimal fill implant in proximal and distal region of the endosteal canal would make it difficult for surgeons to perform implants without breaking the femur [21]. In addition, Massin et al. [21] pointed out that fitting is more crucial than filling which indicates that the implant might come into contact with the endosteal to a certain degree, while not necessarily filling the canal. Our study showed that the newly designed stem fitted 42.90% proximally, and in total fitted 53.70% of the endosteal canal. Engh et al. [45] highlighted the success rate of straight stems compared to anatomic stems with anterior metaphyseal bow which fills the endosteal canal in sagittal view. They correlated the radiographic success rate with straight stem filling from a frontal view [45]. Our study demonstrated the stem filling the endosteal canal to 83.10% at frontal view and 71.70% at lateral view, which would provide better initial fixation. Furthermore, the straight stems eradicated the femur sides demand for left and right femora [8,23]. However, Fukui Medical School (FMS) [20] had introduced a different approached whereby they began designing the straight cementless stem (FMS), followed by an anatomical stem anterolaterally flared [13,14]. They claimed that the anatomical stem produced better results in terms of fit and fill, stress distribution and micromotion [13]. As well, Massin et al. [45] emphasized that the undersized implant in frontal and sagittal view would produce lower fixation stability, and the implant should be modified either at the metaphyseal or distal region frontally. In our study, we have optimized the implant in accordance to the morphology study done prior to the intramedullary canal in frontal and sagittal view. We believe that the optimal contact area between stem and endosteal would enhance osseointegration stem – bone, which leads to better fixation stability. The cross section was chosen based on the commercial implants provided, which fitted and filled the canal. This cross section (wedge shape proximally and medially, and circle shape distally) mimicked the actual endosteal, and contributed to stress distribution especially at lateral regions, decreasing implant failure [46,47]. Furthermore, our newly designed stem had lateral flares which provided a wider base and better contact area with lateral cortex at metaphyseal region. Leali et al. [48] proposed the “rest fit” concept of the lateral flare which prevents subsidence, and improves load distribution at proximal region. Walker et al. [35] supported that this feature leads to additional concentric loading proximally and alleviates stress transfer distally. In addition, lateral flared behavior during revision showed significant bone stock preservation [49].

### Nonlinear three dimensional (3D) finite element analysis

Finite element analysis has become an essential tool for researchers to predict the early outcome of implants, the interaction between implant – bone, and to rectify the problems associated with the design. The present study focused on three parameters of the analysis; equivalent von Mises stress, micromotion and displacement. The general issue in stem implantation is stress shielding at the proximal region where bone atrophy occurs due to a lack of stress at the calcar region. Several factors are related to load distribution in cementless implants; stem geometry, stem – bone interface configuration, and osseointegration [13]. We looked at the stress distribution in intact femurs and found the maximum stress was 108.4 MPa as shown in Figure 8. The stress is normally distributed medially and laterally from the point load of muscle configuration, vastus medialis and vastus lateralis, respectively. On the other hand, the stress normally transferred at the proximal region for the newly designed cementless stem is shown in Figure 9. Stair climbing load distribution demonstrates more stress at the metaphyseal region, especially at calcar which subsequently prevents stress shielding. In addition, proximal osseointegration contributed to the stress distribution at stem – bone interface which preserved bone stock at this region [50]. The results also illustrated that the stem maximum stress (65.38 MPa) did not exceed the bone’s yield strength which was 160 MPa, [16,51] and the intact femur. Abdul-Kadir et al. [32] reported a safety factor of 2 to achieve stem stability with low interference fit of 50 mm during surgery using Alloclassic stem. In addition, Senalp et al. [52] demonstrated a safety factor between 2.18 – 3.27 for four different types of stem made by titanium alloy under static analysis. Based on the previous study, our newly designed stem with a safety factor of 2.45 ensured that the implant could not fracture the bone. The cross section geometry, which has mimicked the endosteal canal shape and diameters, aids the stress distributed to the implant. On the other hand, a review by Khanuja et al. [53] found that the success rate for all types of cementless stems are similar despite the diversity of principle stem designs and femur preparation methods. Additionally, they attributed the excellent survival rates to the stem’s geometrical design rather than the choice of material and fixation surface [47,53].

Stem fixation stability during physiological loading and osseointegration between stem – bone interface are crucial factors which influence the implant lifecycle. These two conditions are usually correlated with micromotion and displacement of the stem within the femur canal. The lack of primary fixation stability would contribute to thigh pain and eventual loosening of the stem due to the continuous disturbance during osseointegration [32]. Several studies demonstrated a micromotion threshold of 150 μm; beyond 150 μm fibrous tissue begins to form, while less than 40 μm primarily stimulates bone ingrowth [32,54]. However, our present study showed acceptable micromotion (4.76 μm) and displacement (1.34 μm), which promoted osseointegration at the stem – bone interface especially at the metaphyseal region. The optimal cross section of the newly designed cementless stem and the curvature radius mimicking the femora morphology contributed to primary fixation stability and low micromotion displacement. Viceconti et al. [55] pointed out through their statistical finite element analysis of over 1000 cases that the stem – endosteal canal differed by up to 1 mm at arbitrary locations on the interface and is prone to produce a grossly loosened stem in 2% of patients, while for another 3 – 5% the high level of expected micromotion makes it difficult to avoid any substantial bone formation. Kawahara et al. [13,14] reported excellent FMS stem results with a 99% survival rate as they performed a morphological study of dysplastic Japanese patients in a previous non linear 3D finite element clinical trial. In addition, they verified reduced radiolucency around the femoral stem in the Gruen zone, minimal subsidence, appropriate stress shielding and the promise of medium term stability in their patients [14].

### Limitations

There are several limitations in our present study. Firstly, the anthropometric data were taken by single observer resulting in potential bias. However, based on our previous study on femur morphology [6-9], precautions have been taken whereby all anthropometric data were taken three times and verified by an experienced orthopedic surgeon to minimize the single observer error. For finite element analysis, the stem and bone were assumed to be fully bonded without penetration. In clinical practice, surgeons are allowed to err 50 μm on each side rather than risk the femora fracture [32]. Furthermore, Petterson et al. [11,12] emphasized that the degree of contact pressure interference penetration during implant fixation is difficult to ascertain due to several factors such as the implant’s size, femora size and quality, and force exerted during surgery. In this study, our newly designed stem demonstrated well distributed stress proximally, and micromotion under threshold for osseointegration (less than 40 μm). However, further studies regarding biomechanical testing are required to validate the finite element result before clinical trial. In addition, several stem designs of commercial implants were needed to demonstrate and compare the optimal performance of our newly designed stem. Still, comparison with other cementless stems from the literature showed that our stem is not inferior to established off the shelf implants. Although dynamic loading was applied to the implant in the actual gait cycle, we only simulated static loading in this study. In addition, the “average” femora model was used for both fit and fill analysis, and finite element analysis. This “average” femora model was developed from our previous morphology study [6-9] and represented the local population. Other physiological factors such as age, gender and bone condition were not considered in this study.

## Conclusion

In conclusion, we would like to stress the importance of stem design based on femur morphology, especially in Asians. The cementless stem design was crucial especially at the metaphyseal region which provided initial fixation stability. In addition, a universal stem of variable size is not the only option available to the peculiar morphology of the Asian population. We hope that this new design process framework will shorten the design cycle, and help researchers to design better femoral stems by identifying the major steps which must be taken and providing anthropometric datasets that could be used as guidelines. The use of accurate three dimensional models obtained from morphological analysis and finite element analysis could be used as preclinical assessment tools to mimic the actual optimal conditions, of stem geometry, and to rectify any problems prior to fabrication.

## Competing interests

The authors report no conflicts of interest in this work.

## Authors’ contributions

BMY, SSH, ZAH and LMH were responsible drafting first manuscript and running the experiment. NAM, HARA, MNA and KASA reviewed and revised the manuscript. All of the authors participated in design study and approved the final version of the manuscript.

## Pre-publication history

The pre-publication history for this paper can be accessed here:

http://www.biomedcentral.com/1471-2474/15/30/prepub
